# Calling for the inclusion of psychosocial professionals in the health system in Burundi

**DOI:** 10.4102/jphia.v16i1.840

**Published:** 2025-05-31

**Authors:** Bonaventure Nikoyandoye, Léandre Simbananiye, Annalisa Casini

**Affiliations:** 1 Department of Psychological Sciences, Centre de Recherche et d’Intervention pour le Développement Individuel, communautaire et Social (CRIDIS), University of Burundi, Bujumbura, Burundi; 2 Psychological Sciences Research Institute, Université Catholique de Louvain, Louvain-La Neuve, Belgium; 3 Department of Psychological Sciences, Faculty of Psychology and Educational Sciences, University of Burundi, Bujumbura, Burundi; 4 Faculty of Law, University of Namur, Namur, Belgium

## Introduction

Worldwide, mental health is a high-cost public health concern that affects people of all ages and from all segments of the population. However, in low-income countries, efforts are often concentrated on biomedical care, and the psychosocial axis is neglected. This is reflected in the low number of mental health interventions and the scarce financial and human resources allocated to them. This is true in most sub-Saharan African countries in general and Burundi in particular. This article aims to advocate for a change in the approaches and practices of decision-makers and health professionals to ensure the inclusion of psychosocial care and professionals in the Burundian health system.

## The Burundian mental health landscape

In sub-Saharan Africa, mental disorders, both neurological and substance-related, represent a major, yet growing burden.^1,2^ Burundi is no exception. In this country, because of the periods of socio-political and economic crises accompanied by mass violence that the country has gone through over the last few decades, psychological distress and mental disorders are quite common among the population. In the baseline survey of the programme for the integration of mental health in primary healthcare services carried out by the *Institut de Statistiques et d’Etudes Economiques du Burundi*,^3^ 64.5% of the Burundian population manifested psychological ill-being, while 47.5% of the respondents experienced an episode of severe disorders. The number of people suffering from mental disorders is greater than the number of people being treated. These people rarely come to the care facilities or do so as a last resort. Besides, because of a lack of appropriate training, most primary healthcare workers are not able to detect and treat mental disorders and psychosocial problems. This is illustrated by the number of patients who attended mental healthcare facilities during the 2022 period – over 18 000 patients with conditions ranging from acute psychotic disorders to depression.^4^

Poor resource management, health service overload, and resistance from policymakers and health professionals are the major obstacles to the effective inclusion of mental healthcare in primary and community healthcare and the incorporation of psychosocial professionals.^5^ Indeed, the human resources issue in mental health is characterised by an under-resourced health workforce with uneven geographical distribution and limited skills.^2^

Yet, integrating mental healthcare into primary healthcare would provide multiple benefits. First of all, this integration would reduce the gap between need and access to care.^5^ People would be able to access mental health services at a lower cost and remain close to their homes. They would maintain their connection to their families and would not deviate from their daily activities. Moreover, this kind of care for mental health and psychosocial problems would foster mental health promotion in the community. Finally, it would facilitate the care and monitoring of people affected, who, at the same time, would also avoid indirect costs associated with specialised care in remote areas.

Given that psychosocial problems are the source of a significant emotional burden for the people concerned and their families and create economic and social difficulties that affect the whole society, the provision of psychosocial personnel would be an important asset in helping to resolve these problems and would reduce the indirect costs that affect the patients, their families and the national economy.^5^ In addition, psychosocial professionals’ interventions would also help address the issue of discrimination. Indeed, in Burundi, most people with mental disorders are poor and unsupported. They are homeless and do not receive the treatment and care they need. These people are discriminated against and stigmatised by society in the spheres of education, employment and community activities, preventing them from participating in social, economic and political life.

To better understand the underlying reasons for this situation, it is necessary to take a closer look at the guidelines for health professionals, health and human resources policies in Burundi, and the place of psychosocial professionals in the Burundian health system.

## Directives of the authorities concerning health professionals

Since the Alma-Ata Conference in 1978 and the drafting of the Ottawa Charter in 1986, the World Health Organization (WHO) has recommended that every country move from medical structures to services for individuals and promote user and carer policies.^5^ Specifically, the WHO advocates against discrimination and stigmatisation, as well as the defence of users’ rights, intersectoral work, social inclusion and access to care for all.^6^ This implies allocating sufficient and qualified human resources to achieve all these objectives.

The weakness of health and especially mental health systems in the African region contrasts with the heavy epidemiological burden because of mental, neurological and substance use disorders.^7^ A major challenge is the shortage of human resources for mental health, which is characterised by an inadequate number of health workers with limited or inadequate skills, poor geographical coverage and unevenly distributed specialists. The WHO estimates that, in this area, there is less than one mental health worker per 100 000 population,^2^ and Burundi is no exception in this respect.

The Burundian health system is structured around three concentric levels.^8^ The central level includes the minister’s cabinet, a permanent secretariat, four General Directorates, three specialised institutions, eight Directorates related to eight health programmes and associated services, and seven national hospitals. This first level is responsible for formulating health policy, strategic planning, coordination, mobilisation and allocation of resources, as well as monitoring and evaluation. The intermediate level comprises 18 provincial health offices (PHOs) and five regional hospitals. It is responsible for the coordination of all health activities in the province. It supports the health districts and ensures good intersectoral collaboration. The third peripheral level includes 49 health districts, 52 hospitals and 1205 health centres, of which 669 are public, 348 private, 147 religious and 41 associative. The health district is the operational unit of the healthcare system. It gathers from the basis to the top, the community level, the health centres (HC), the municipal hospitals (first reference) and the district hospital (second reference). The involvement of the communities in the healthcare system is supposed to take place through the management of the health centres via the health committees (i.e. *Comités de Santé* or COSA) and the health management committees (i.e. *Comités de gestion de santé* or COGES) of the HCs. The communities are represented by the community health agents (CHAs), who are grouped into Community Health Agent Groups (CHAGs). Community health agents collaborate with health centre providers, community actors, local leaders and families.^9^

Officially, the government shows a clear commitment to tackling the existing mental health problems that represent a serious challenge for the health system. Indeed, mental health is considered an intervention priority in the integrated strategic plan for the fight against non-transmissible diseases included in the National Health Development Plan (PNDS III 2019–2023). The plan provides more explicitly for three specific strategic axes, namely, the integration of mental healthcare in healthcare structures, the development of community interventions in mental health and psychosocial care, and the information, education and communication in mental health.^10^

## Health policies in Burundi and human resources

According to the Ministry of Public Health AIDS Control,^11^ the General Directorate of Health Services is responsible for ensuring horizontal integration of vertical programmes across the entire health service network. To achieve this, the General Directorate must develop ‘interrelationships, complementarities, and synergies between departments and services at different health system levels’. Government agencies, civil society organisations and supporting health agencies should also be involved in this integration exercise, starting with the technical integration of health programmes and activities from all health programmes and projects. The Human Resources Department ensures that staff integration and equitable distribution across the territory are established and respected.

Furthermore, the Burundian government has made efforts to describe job profiles and map the human resources providing services in the public and private sectors, including mental health, with regard to the management of human resources in general and mental health human resources in particular. In addition, the Republic of Burundi has just issued a decree on the creation, organisation and operation of the Burundi Order of Allied Health Professions (*Ordre des professions alliées de la santé du Burundi*, OPASBU), which includes clinical psychologists.^12^

Despite these efforts, difficulties remain. The implementation of human resources management reference documents has not yet been completed, and the human resources management software is still awaited, even though it is expected to contribute to the non-decentralisation of human resources management. Similarly, a human resources observatory has been set up but is still not operational.

## The position of psychosocial professionals in the health system in Burundi

At the community level, mental health issues and physical health are inseparable. Most patients who come to the health centre suffer from both physical and mental problems. Integrated primary care services should then ensure that patients are treated with a holistic approach. This would help address jointly the mental health needs of individuals with physical disorders and the physical health needs of individuals with mental disorders. Therefore, when considering the integration of mental health into healthcare at the health centre and in the community, it is essential to develop strategies for enhancing the awareness and the skills of providers of preventive, curative, promotive and adaptive mental healthcare.

According to national guidelines^9^ and the national health standards of Burundi’s Ministry of Public Health and AIDS Control,^13^ mental health services need to be set up from first-referral hospitals to national referral hospitals. The plan foresees the creation of multidisciplinary teams comprising a specialist doctor, a general practitioner trained in mental health, a clinical psychologist, a nurse trained or specialised in mental health and a social worker.

Following the guidelines, health professionals must work together as a multidisciplinary team, carrying out tasks such as psychological and psychiatric consultations, diagnosis, treatment and follow-up of cases, referral and counter-referral, psychosocial support, listening, advice and guidance, supportive psychotherapy, therapeutic education, short-term hospitalisation and liaison consultation for patients hospitalised in other departments. Of course, these psychosocial specialists can also contribute to the ongoing training and supervision of hospital staff (HS) and district hospital staff.

To implement these guidelines, it is proposed to train at least two people per hospital, from first reference hospitals to national hospitals, to work in the emergency and internal medicine departments. These departments are singled out because they have patients with excessive psychological distress.

However, the figures available show that the resources allocated until December 2021 and those needed to implement the national guidelines for integrating mental healthcare into the health system in Burundi are still well below expectations.^8,14^ Indeed, the expected workforce of the health centre is not fully staffed and is insufficiently trained to carry out these activities. More precisely, by 2021, the Burundian health system will be 63.5% short of the recommended workforce. That is, except for nurses and general practitioners, all professional categories show a gap of well over 50%. Indeed, out of a total of 11 768 community health agents (CHAs), only 3679, corresponding to a shortfall of 68.74%, have been trained in the prevention and control of mental illness. Community health agents, who work with local community associations, local leaders, the mentally ill and their families, are elected by the community on a self-nomination basis. By law, candidates are eligible if they have completed the primary cycle (Grade 9); but because of the lack of candidates, a person who has completed Grade 6 may also be considered.^8^ Community health agents work as volunteers. They provide community health promotion services in collaboration with local associations, local leaders and families. In the area of psychosocial and mental health, activities include information, education and communication (IEC), awareness raising, patient identification and referral, family support and referral, early detection of disorders, psychosocial counselling and support, home visits, provision of psychosocial support in situations of psychological distress and socio-economic reintegration of families (e.g. income-generating activities, etc.).^9^

The health promotion technicians (HPTs) trained in the prevention and control of mental illnesses, who should be assigned to all healthcare structures and act as liaisons with the HCs and CHAGs are only 361, corresponding to 39.28% of the recommended staff requirement. This leaves a shortfall of 548 HPTs with different levels of specialisation. Likewise, there are 95 social workers available, corresponding to 11.05%. The shortage is 754, although they are supposed to be assigned to all levels of care. Moreover, there are only 15 clinical psychologists, representing 7.89%, against the 190 needed. These figures are even more concerning given the crucial role psychosocial professionals play in care structures, a fact that is already well-established.

According to the databases of the Ministry of Public Health and AIDS Control in the Directorate of Human Resources for Health, the situation has barely evolved between 2016 and 2021. The number of psychologists decreased from 52 to 15; social workers remained at the same number, namely 32; and health promotion technicians saw their numbers increase by 7, going from 354 to 361. With regard to the biomedical staff categories, the numbers have increased slightly: doctors were 647 in 2016, and in December 2021, they are 717; nurses have increased from 7332 to 7714; the number of anaesthetists, midwives, laboratory technicians, and radio technicians has also increased. It is thus worth asking why the number of psychosocial staff tends to decrease, while the number of biomedical staff tends to increase. Psychological staff who leave are not being replaced, and there is no new recruitment, even though young graduates from universities are available for these positions.

More fundamentally, there is still a major shortage of staff, in terms of both quantity and quality, particularly in the psychosocial professions, while existing human resources are unevenly distributed or underused.^4^ Nonetheless, training institutions for professionals in the psychosocial field do exist and produce sufficient human resources; however, these resources are still not being deployed. Indeed, in Burundi, six universities have psychology faculties, graduating 1309 students in psychology during the 2019–2020 academic year alone. Additionally, two health institutes – the National Institute of Public Health and the Ecole Supérieure des Sciences de la Santé – offer mental health and psychiatry courses, training 93 students.

It is true that economic constraints and misconceptions about professionals’ skills often hinder this process of improving patient care. However, political decision-makers and hospital managers are not always aware of the role psychologists can play in alleviating patient distress.

## Discussion

Joining other scholars who have already advanced similar arguments before us,^15,16^ this article calls for a change in the approaches and practices of political decision-makers, hospital managers and healthcare professionals in Burundi so that psychosocial care and professionals can be integrated into the healthcare system. Health human resources, and psychosocial staff, in particular, have been the focus of our attention. Psychosocial staff play a fundamental role at all levels of the health system. From the community to the national level, their services complement those of other staff. They are mainly involved in raising awareness among the population and especially the community, providing support and guidance for families, early detection of disorders, combating stigmatisation and discrimination, psychosocial support in situations of psychological distress, and the socio-economic reintegration of families and patients who have stabilised and/or recovered. At the level of care structures, psychosocial factors play a role in lifestyle changes, emotional regulation and the development of prevention, promotion, adaptation and rehabilitation programmes, as well as community rehabilitation.

Psychosocial staff are diverse regarding their initial training, tasks and place of employment. The transition between the psychiatric hospital, the general hospital and the community calls on everyone to readjust their approach.

The traditional view of mental illness and its representations, which leads health professionals to focus on the illness and not on the person, and to attribute all responsibility to the illness and remove all responsibility from the patient, needs to be broadened. Beyond treating illnesses, psychosocial staff play a crucial role in preventative mental healthcare. For example, psychologists are trained to help patients adopt healthy lifestyles and teach emotional regulation strategies, which can reduce the risk of mental health issues recurring or worsening. They are also involved in developing prevention programmes. When these programmes are implemented from an early age, they can help reduce the onset of physical disorders in adulthood.

Notwithstanding the new national guidelines for integrating mental health into primary healthcare and the national standard norms, the number of staff responsible for the psychosocial health of the population remains well below the requirements. According to the 2020 statistical yearbook,^4^ there were only 0.12 psychologists, 0.03 psychiatrists and 0.77 social workers per 100 000 inhabitants in the country’s healthcare system. This echoes the alarming estimates made by the WHO^17^ at the sub-Saharan African level. Only professionals who have been working in the health sector for a long time, such as general practitioners, trained nurses, HPTs and CHAs, have a score of more than 1 per 100 000 inhabitants. With the new decree governing the Order of Paramedical Professions, which includes psychologists, the hope that psychosocial professionals will be recruited and properly integrated into the healthcare system is beginning to take shape.

Taking these considerations into account, a strategic focus on the integration of psychosocial professionals is needed. This will make it possible to design a human resources development plan over a chosen period (3 years or 5 years). Yet, the efforts of the health sector alone are not enough to establish universal health coverage, which is a set of reforms that must be carried out in several sectors at the same time (health, social protection, finance, education, justice, sectors in charge of health determinants, etc.).

The Ministry of Public Health and AIDS Control should use this work as a basis for planning human resources in the field of mental health and psychosocial support. The available data should be used to draw up a recruitment plan for the implementation of the national guidelines on integrating mental health into primary healthcare. In addition to assessing the profiles and numbers of these human resources, it is essential to look at their motivation, retention and loyalty. Furthermore, it would be beneficial to identify the needs of psychosocial professionals, as well as the challenges they identify and the solutions they propose to improve their effectiveness and their integration into the Burundian healthcare system. Ultimately, their successful integration into the healthcare system would facilitate its functioning, improve the quality of patient care and alleviate the excessive burden on all healthcare staff.
